# Genetic variant rs4072037 of *MUC1* and gastric cancer risk in an Eastern Chinese population

**DOI:** 10.18632/oncotarget.7527

**Published:** 2016-02-20

**Authors:** Li-Xin Qiu, Rui-Xi Hua, Lei Cheng, Jing He, Meng-Yun Wang, Fei Zhou, Xiao-Dong Zhu, Meng-Hong Sun, Xiao-Yan Zhou, Jin Li, Ya-Nong Wang, Ya-Jun Yang, Jiu-Cun Wang, Li Jin, Wei-Jian Guo, Qing-Yi Wei

**Affiliations:** ^1^ Department of Medical Oncology, Fudan University Shanghai Cancer Center, Department of Oncology, Shanghai Medical College, Fudan University, Shanghai, China; ^2^ Cancer Institute, Collaborative Innovation Center for Cancer Medicine, Fudan University Shanghai Cancer Center, Shanghai, China; ^3^ Department of Oncology, The First Affiliated Hospital of Sun Yat-Sen University, Guangzhou, China; ^4^ Department of Pediatric Surgery, Guangzhou Women and Children's Medical Center, Guangzhou Medical University, Guangzhou, Guangdong, China; ^5^ Department of Pathology, Fudan University Shanghai Cancer Center, Shanghai, China; ^6^ Department of Gastric Cancer and Soft Tissue Sarcoma Surgery, Fudan University Shanghai Cancer Center, Shanghai, China; ^7^ Ministry of Education Key Laboratory of Contemporary Anthropology and State Key Laboratory of Genetic Engineering, School of Life Sciences, Fudan University, Shanghai, China; ^8^ Fudan-Taizhou Institute of Health Sciences, Taizhou, Jiangsu, China; ^9^ Duke Cancer Institute, Duke University Medical Center and Department of Medicine, Duke University School of Medicine, Durham, NC, USA

**Keywords:** gastric cancer, genetic susceptibility, MUC1, polymorphism

## Abstract

Published data on the association between the *MUC1* rs4072037A > G polymorphism and gastric cancer (GCa) risk were inconclusive. To derive a more precise estimation of the association, we conducted a large GCa study of 1,124 cases and 1,192 controls to confirm this association in an Eastern Chinese population. Our results showed that the G allele was strongly associated with a decreased GCa risk in the study population [GG vs. AA, odds ratio (OR) = 0.47, 95% confidence interval (CI) = 0.31–0.73; AG/GG vs. AA, OR = 0.82, 95% CI = 0.68–0.99; GG vs. AA/AG, OR = 0.48, 95% CI = 0.32–0.74]. These associations remained significant in subgroups of age, tumor site, drinking and smoking status. Moreover, this association was supported by an additional meta-analysis of published studies. In summary, these results suggest that the *MUC1* rs4072037G allele may be a low-penetrating protection factor for GCa risk in Chinese populations.

## INTRODUCTION

Gastric cancer (GCa) is one of the most common cancers and one of the leading causes of cancer-related deaths in the world. There were 951,600 new GCa cases and 723,100 deaths in 2012, accounting for 8% of the cancer cases and 10% of cancer deaths, respectively [1]. Therefore, GCa is a major public health problem whose mechanism of carcinogenesis is still not fully understood. It is well-known that environmental factors and low-penetrance susceptibility genes may be important in the etiology of GCa. For example, a higher rate of *Helicobacter pylori* (*HP*) infection (70–90%) in developing countries than in developed countries (25–50%) may have increased GCa risk in developing countries [2, 3]. However, not all *HP* carriers will develop GCa, suggesting that other factors are also important in the etiology, such as tobacco smoking, alcohol use and dietary habits [4]. In addition, genetic factors for the GCa risk are important as well, because the success in identifying at-risk populations by associations between genetic variants and GCa risk is encouraging [5–8]; however, it is necessary to confirm those genetic factors that have been reported to be important in the etiology of GCa.

**Figure 1:**
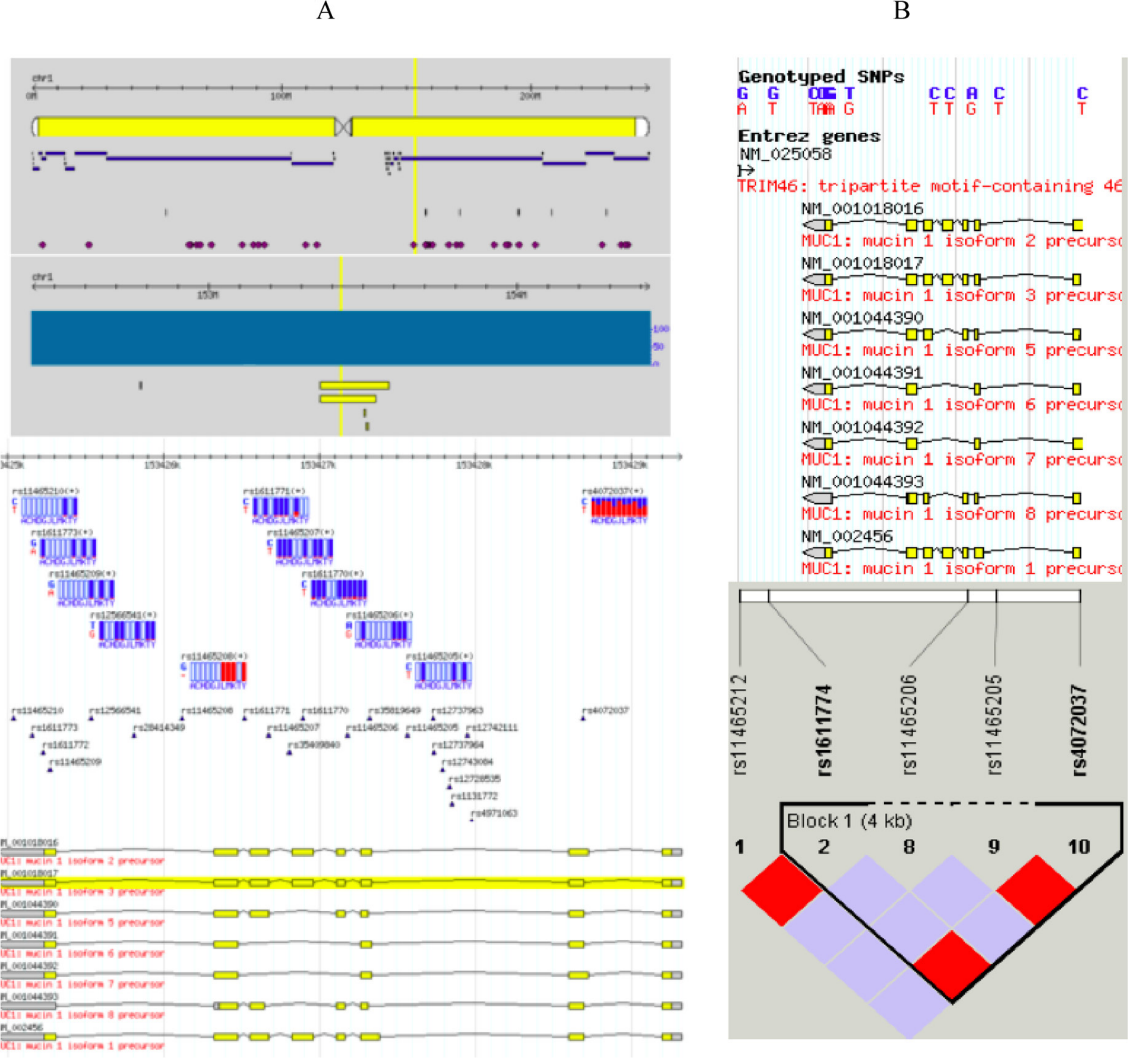
Reported *MUC1* SNPs Although MUC1 has 712 SNPs as reported to the dbSNP database, only 11 SNPs **(A)** with genotypes are actually confirmed in the HapMap database, of which only rs4072037 has a MAF > 0.05, representing a block of 4 SNPs **(B)**.

The *Mucin 1* (*MUC1*) gene is a member of the mucin family encoding membrane-bound glycoproteins. The mucin 1 protein protects gastric epithelial cells from a variety of external insults that potentially cause inflammation, leading to carcinogenesis. Although *MUC1* has 712 SNPs as reported to the dbSNP database (http://www.ncbi.nlm.nih.gov/projects/SNP/snp_ref.cgi_showRare=on&chooseRs=all&go=Go&locusId=4582), only 11 SNPs (Figure 1A.) have actually been confirmed in the HapMap database, of which only rs4072037 has a MAF > 0.05, representing a block of 4 SNPs (Figure 1B). The rs4072037 A > G polymorphism is located in the 5′ untranslated region (UTR) of the second exon of *MUC1* at chromosome 1q22, alters transcriptional regulation, and determines splice variants in *MUC1* [9]. Several studies reported an association between the *MUC1* rs4072037 A > G polymorphism and GCa risk [10–16], but the results were inconclusive, especially different by ethnic group and primary tumor site. To further confirm this reported association, we conducted a replication study in an Eastern Chinese population with a relatively larger sample with subgroup analysis. Furthermore, a meta-analysis was also performed to further validate the association.

## RESULTS

The characteristics of participants included in this hospital-based case-control study were described elsewhere [17], but one sample in cases and four samples in controls failed to be genotyped in the current study. Thus, the final analysis included 1,124 GCa cases and 1,192 cancer-free controls. Participants were well matched by age and sex with more smokers and drinkers in the controls, but these variables were further adjusted in the following multivariate analysis.

Table 1 lists allele frequencies of the rs4072037 A > G SNP in cases and controls and the estimated association between this SNP and GCa risk. Overall, the G allele was associated with a decreased GC risk in the study population [GG vs. AA, OR = 0.47, 95% CI = 0.31–0.73; AG/GG vs. AA, OR = 0.82, 95% CI = 0.68–0.99; GG vs. AA/AG, OR = 0.48, 95% CI = 0.32–0.74]. In the stratified analysis (Table 2), these associations remained significant in subgroups of age, tumor site, drinking and smoking status.

**Table 1:** Logistic regression analysis of associations between the genotypes of MUC1 rs4072037 A > G and gastric cancer risk in an Eastern Chinese population

Genotype	Cases (*N* = 1, 124)	Controls (*N* = 1, 192)	*P*^a^	Crude OR (95% CI)	*P*	Adjusted OR (95% CI)^b^	*P*^b^
*MUC1* rs4072037							
AA	852 (75.8)	854 (71.6)	0.002^c^	1.00		1.00	
AG	240 (21.4)	270 (22.7)		0.89 (0.73–1.09)	0.253	0.91 (0.74–1.11)	0.328
GG	32 (2.8)	68 (5.7)		0.47 (0.31–0.73)	0.0006	0.47 (0.31–0.73)	0.0007
AG/GG	272 (24.2)	338 (28.4)	0.023^d^	0.81 (0.67–0.97)	0.023	0.82 (0.68–0.99)	0.035
Additive				0.79 (0.68–0.92)	0.002	0.80 (0.68–0.93)	0.003
AG/AA	1092 (97.2)	1124 (74.2)		1.00		1.00	
GG	32 (2.8)	68 (5.7)	0.0007^e^	0.48 (0.32–0.74)	0.0009	0.48 (0.32–0.74)	0.0009

CI, confidence interval; OR, odds ratio.

a Chi square test for genotype distributions between cases and controls.

b Adjusted for age, sex, smoking and drinking status in logistic regression models.

c for additive genetic models.

d for dominant genetic models.

e for recessive genetic models.

**Table 2:** Stratification analysis for the association between *MUC1* rs4072037 A > G polymorphism and GC risk in an Eastern Chinese population

Variables	rs4072037 (cases/controls)		Crude OR (95% CI)	*P*	Adjusted OR^a^ (95% CI)	*P*^a^
	AG/AA	GG				
Median age, yr						
≤ 59	564/574	14/32	0.45 (0.24–0.84)	0.013	0.45 (0.24–0.85)	0.014
> 59	528/550	18/36	0.52 (0.29–0.93)	0.027	0.51 (0.28–0.91)	0.023
Sex						
Males	779/773	21/52	0.40 (0.24–0.67)	0.0005	0.40 (0.24–0.68)	0.0006
Females	313/351	11/16	0.77 (0.35–1.69)	0.515	0.81 (0.37–1.78)	0.591
Smoking status						
Never	664/574	21/32	0.57 (0.32–1.00)	0.048	0.55 (0.31–0.98)	0.041
Ever	428/550	11/36	0.39 (0.20–0.78)	0.008	0.40 (0.20–0.81)	0.010
Pack-year						
0	664/574	21/32	0.57 (0.32–1.00)	0.048	0.55 (0.31–0.98)	0.041
≤ 25 (mean)	222/332	5/21	0.36 (0.13–0.96)	0.041	0.36 (0.13–0.97)	0.044
> 25 (mean)	206/218	6/15	0.42 (0.16–1.11)	0.081	0.48 (0.18–1.27)	0.137
Drinking status						
Never	828/804	26/44	0.57 (0.35–0.94)	0.028	0.57 (0.35–0.94)	0.027
Ever	264/320	6/24	0.30 (0.12–0.75)	0.010	0.31 (0.12–0.77)	0.011
Tumor site						
GCA	297/1124	8/68	0.45 (0.21–0.94)	0.033	0.42 (0.20–0.89)	0.024
NGCA	795/1124	24/68	0.50 (0.31–0.80)	0.004	0.50 (0.31–0.81)	0.005

a Adjusted for age, sex, smoking and drinking status in logistic regression models.

**Figure 2:**
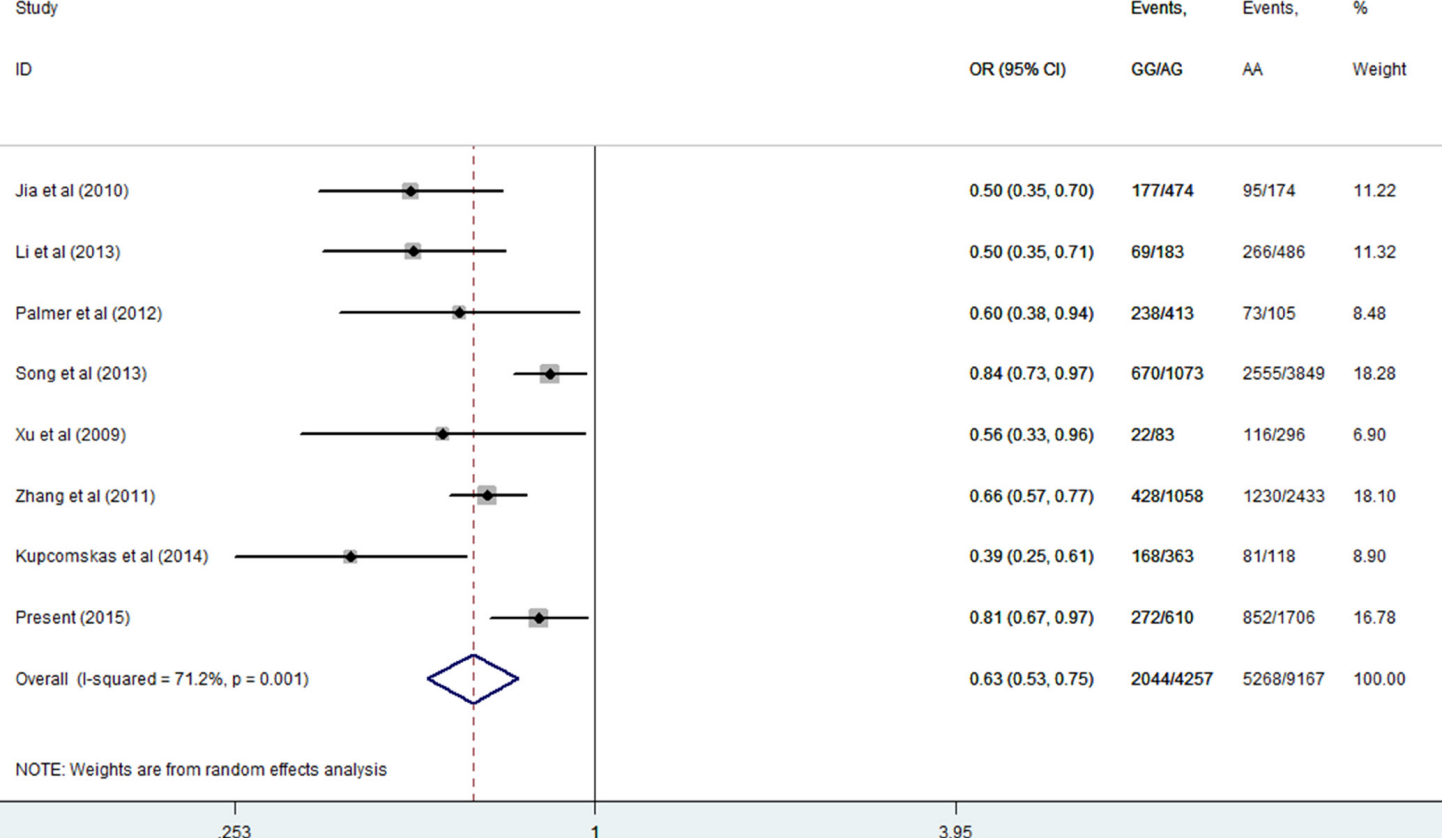
Meta-analysis for the association between *MUC1* rs4072037 SNP and GCa risk in the dominant genetic model (random-effects model)

**Figure 3:**
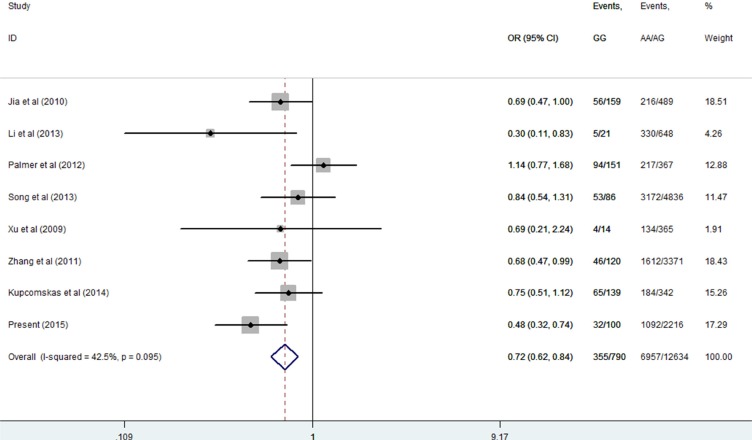
Meta-analysis for the association between *MUC1* rs4072037 SNP and GCa risk in the recessive genetic model (fixed-effects model)

Then, we performed a mini meta-analysis, including the present study, of eight studies with 7312 cases and 6112 controls [10–16]. The pooled data indicated that the G allele was strongly associated with a decreased GCa risk (Table 3: AG vs. AA: OR = 0.64, 95% CI = 0.54–0.77; GG vs. AA: OR = 0.55, 95% CI = 0.46–0.65; AG/GG vs. AA: OR = 0.63, 95% CI = 0.53–0.75, Figure 2; and GG vs. AA/AG: OR = 0.72, 95% CI = 0.62–0.84, Figure 3) without significant publication bias. However, significant heterogeneities across studies were present in these genetic models. Thus, we performed a sensitivity analysis to assess the effects of each study on the pooled results. The pooled ORs were not affected by omitting each of studies at a time (data not shown), which suggests that the overall results are robust.

**Table 3:** Meta-analysis for the association between the *MUC1* rs4072037 A > G SNP and GCa risk

Genotype	No. of Studies	No. of Cases	No. of Controls	OR (95% CI)	*P*	*P*_heterogeneity_	*I*^2^ (%)	Model
*MUC1* rs4072037								
AG vs AA	8	6957	5677	0.64 (0.54, 0.77)	P < 0.001	0.001	71.0%	Random
GG vs AA	8	5623	4334	0.55 (0.46, 0.65)	P < 0.001	0.239	23.8%	Fixed
AG/GG vs AA	8	7312	6112	0.63 (0.53, 0.75)	P < 0.001	0.03	8 71.2%	Random
GG vs AA/AG	8	7312	6112	0.72 (0.62, 0.84)	P < 0.001	0.095	42.5%	Fixed

## DISCUSSION

In addition to environmental and lifestyle factors for GCa risk, genetic factors are also important in identifying at-risk populations for primary prevention of GCa. The results presented here consistently showed that the G allele of the *MUC1* rs4072037 A > G SNP was associated with a decreased risk of GCa. This SNP located in the 5′ UTR of the second exon is predicted to have an effect on the splicing of the primary transcripts, which in turn determines the type of variants. Studies suggest that the G allele results in the expression of variant 2, while the A allele results in the expression of variant 3 [9, 24]. The structural difference between these two variants leads to insertion/deletion of nine amino acids encoded by the second exon, which are involved in the N-terminal signal peptide. This differential signal peptide may lead to a different function of the encoded variant protein. Also, the A allele reduces the transcriptional activity, which may result in decreased MUC1 expression [9]. Furthermore, MUC1 can block the adhesion of *HP* blood group antigen-binding adhesion and sialic acid-binding adhesion to the gastric mucosa, which in turn limits the *HP* colonization [25, 26], and MUC1 acts as a barrier against exogenous insults in normal epithelial cells [25]. Therefore, low expression of MUC1 may cause a reduction in its barrier function in the stomach and subsequently increases GCa susceptibility. Such a hypothesis needs to be tested in additional mechanistic studies.

There are some limitations in the present study. First, although age, sex, smoking and drinking status, and tumor site were taken into consideration for subgroup analysis, other important risk factors, such as diet and HP infection, were missing in the study, which might also contribute to the etiology of GCa. Second, new classification of GCa tumor types, which was not available for the patients diagnosed years ago, is also important and may have a different genetic basis in the etiology. Third, the sample size of the cases in subgroups was largely reduced in the stratification analysis, which may lead to limited statistical power in subsequent analysis.

In conclusion, the present study confirmed that the G allele of the *MUC1* rs4072037 SNP was a low-penetrating protection factor for GCa risk. However, future studies should incorporate diet, *HP* infection status and Lauren classification to better understand the associations between the *MUC1* rs4072037 SNP and GCa susceptibility.

## METHODS

### Study subjects

This study included patients who were recruited from our ongoing molecular epidemiology study of GCa, and the cases and controls were described previously [17–19]. Briefly, 1,125 unrelated ethnic Han Chinese patients with newly diagnosed and histopathologically confirmed primary gastric cardia adenocarcinoma and non-gastric cardia adenocarcinoma (NGCA) were recruited from Fudan University Shanghai Cancer Center (FUSCC) in Eastern China between January 2009 and March 2011. Patients other than histopathologically confirmed primary GCa were excluded. In addition, 1,196 age and sex-matched cancer-free ethnic Han Chinese controls were recruited from the Taizhou Longitudinal (TZL) study conducted at the same time period in Eastern China as described previously [20]. Blood samples from both GCa patients and cancer-free controls were provided by the tissue bank of FUSCC and the TZL study, respectively. All participants had signed a written informed consent for donating their biological samples to the tissue bank for scientific research. Demographic data and environmental exposure history of each participants were collected. The overall response rate was approximately 91% for cases and 90% for controls. This research protocol was approved by the FUSCC institutional review board.

### SNP genotyping

According to a relevant protocol, we extracted genomic DNA from peripheral blood. The rs4072037 SNP was genotyped by the TaqMan assay with ABI7900HT real-time PCR system (Applied Biosystems) as reported previously [17]. Participants’ status was unrevealed in the genotyping process. As recommend by the company, four negative controls (without DNA templates) and two duplicated samples were included in each 384-plate for the quality control. The assays were repeated for 5% of the samples, and the results were 100% concordant.

### Statistical methods

The *χ*^2^ test was used to assess differences in the distributions of demographic characteristics between cases and controls. The association between SNP and GCa risk was assessed by odds ratio (OR) and 95% confidence intervals (CIs) in heterozygous (AG vs AA), homozygous (GG vs AA), dominant (AG + GG vs AA), recessive (GG vs AG + AA), and additive (G vs A) models, respectively. OR values were calculated by both univariate and multivariate logistic regression models. Moreover, logistic regression tests for each genetic model were adjusted for age, sex, drinking and smoking status. Furthermore, the association between the *MUC1* rs4072037 SNP and GCa risk was also stratified by age, sex, smoking or drinking status, and primary tumor site. All statistical process above was achieved by SAS software (version 9.1; SAS Institute, Cary, NC)

To validate the results of the present study, we performed a mini meta-analysis with studies searched from Medline, PubMed and Embase. After using the search terms and inclusion and exclusion criteria as described in previous studies [21, 22], all primary reports were carefully reviewed, and the relevant references in these papers were also searched and reviewed by two independent authors. Then, data were retrieved from the reported studies and pooled crude ORs for heterozygous, homozygous, dominant, and recessive models were calculated. Heterogeneity between studies was estimated by Chi-square-based *Q* test. Pooled ORs were calculated by a fixed- or random-effects model, depending on the heterogeneity between searched studies [23]. To validate the stability of the pooled results and to identify the sources of heterogeneity, the leave-one-out sensitive analysis was performed. Publication bias was shown by the funnel plot, in which the asymmetry was estimated by the Egger's liner regression test, where the statistically significant publication bias was tested, when *P* < 0.05 determined by the *t* test as suggested by Egger. All statistical process was achieved by STATA version 10.0 (Stata Corporation, College Station, TX).
